# The Pseudo-Bilayer Bulk Heterojunction Active Layer of Polymer Solar Cells in Green Solvent with 18.48% Efficiency

**DOI:** 10.3390/polym17030284

**Published:** 2025-01-22

**Authors:** Jingyue Cao, Zheng Xu

**Affiliations:** 1 Key Laboratory of Luminescence and Optical Information, Beijing Jiaotong University, Ministry of Education, Beijing 100044, China; 17118456@bjtu.edu.cn; 2 Institute of Optoelectronics Technology, Beijing Jiaotong University, Beijing 100044, China

**Keywords:** polymer solar cells, pseudo-bilayer bulk heterojunction, morphological evolution, vertical phase separation, green solvent

## Abstract

Planar heterojunction (PHJ) is employed to obtain proper vertical phase separation for highly efficient polymer solar cells (PSCs). However, it heavily relies on the choice of orthogonal solvent in the production process. Here, we fabricated a pseudo-bilayer bulk heterojunction (PBHJ) PSC with cross-distribution in the vertical direction by preparing two layers of PM6 and BTP-eC9 blends in an *o*-XY solution with different dilution ratios to study the morphological evolution of PBHJ film. We found that the PBHJ film exhibits more uniform and suitable continuous interpenetrating network morphology and proper phase separation in the vertical direction for the formation of p-i-n structure. This provides an effective channel for exciton dissociation and charge transport, which is confirmed by both exciton generation simulations and charge dynamics measurements. The PBHJ devices can effectively inhibit trap recombination and accelerate charge separation and transfer. Based on good active layer morphology and balanced charge mobility, all-green solvent-processed PSCs with champion power conversion efficiencies (PCEs) of 18.48% and 16.83% are obtained in PM6:BTP-eC9 and PTQ10:BTP-eC9 systems, respectively. This work reveals the potential mechanism of morphological evolution induced by the PBHJ structure and provides an alternative approach for developing solution processing PSCs.

## 1. Introduction

The power conversion efficiency (PCE) of single-junction polymer solar cells (PSCs) has reached 20%, making breakthrough progress over recent years because of the renewal of highly efficient non-fullerene acceptor materials [1,2]. The mainstream PSCs usually adopt the binary bulk heterojunction (BHJ) architecture, in which the spontaneous phase separation of the electron donor and acceptor provides sufficient donor/acceptor interface for exciton dissociation and forms interpenetrating nanonetwork channels to transport charge [3,4]. As a result, the BHJ structure with ideal morphology has paved the way for the development of high-performance PSCs in recent years. Unfortunately, the random mixing of donor and acceptor inevitably leads to many defects in BHJ films. In particular, the size of the donor/acceptor domain strictly limits the decay of excitons larger than the diffusion length before reaching the donor/acceptor boundary, leading to bimolecular recombination [5,6]. On the other hand, due to poor connectivity in the vertical direction, free charges are easily trapped in isolated structural domains to induce amorphous recombination [7,8]. Some groundbreaking work has proposed that the planar heterojunction (PHJ) structure using layer-by-layer (LbL) processing is inhibited by bimolecular recombination and reduced trap state due to charge transport of neat donor or acceptor phase [9,10]. As a result, the PHJ structure PSCs have also recently made rapid progress [11]. In principle, the PHJ active layer should be formed with orthogonal solvents to ensure that the upper layer deposition does not erode the underlying film. Therefore, the balance between active layer thickness and exciton diffusion length in certain LbL processing is still difficult. Qiang Peng et al. developed the interdigitated heterojunction (IHJ) by extending the PHJ concept, and the vertically arranged donor nanocolumns were wrapped via acceptor to achieve an ordered vertical phase structure [12]. Gang Li’s team reported a guest polymer-tailored LbL strategy that can obtain pre-aggregation of non-fullerene acceptors for constructing the superior structure of the built-in interpenetrating network to reduce trap state and energy loss [13]. It is not difficult to see that the structure optimization of the active layer is crucial to promoting efficient and stable PSCs.

In addition, the most highly efficient BHJ PSCs currently are fabricated using harmful halogenated solvents. Compared with solution processing PHJ PSCs, non-halogenated solvents are gradually being used to replace halogenated solvents because of the need to use orthogonal solvents. Yiwang Chen’s team used the large polarity differences between *o*-xylene (*o*-XY) and tetrahydrofuran (THF) to process the donor and acceptor solutions, respectively, and fabricated highly efficient PHJ PSCs treated with all green solvents [14]. He Yan’s research group reported that a volatile processing aid induces the pre-formation of nanoscale polymer fibers, then reporting a spin-coating toluene (Tol) solution to intertwine with the polymer fibers, resulting in non-halogenated solvent-processed PSCs with a PCE of more than 19% [15]. Chen Xie and collaborators even reported using a donor-containing water-based nanoparticle ink-assisted LbL process to prepare PPHJ structures to optimize vertical phase separation [16]. Therefore, the green solvent-treated LbL processing method is an effective strategy to achieve proper phase separation. However, due to entropic effects, small molecule acceptors have higher solubility than polymer donors in non-halogenated solvents and tend to over-aggregate, making small molecule acceptors difficult to achieve morphological stability and high charge mobility. In contrast, pseudo-bilayer bulk heterojunction (PBHJ) structures have the advantage of complementary BHJ and PHJ to address this issue, first proposed by Yingping Zou’s team [17]. For a favorable vertical phase separation, the donor should be closer to the anode and the acceptor closer to the cathode, while forming an ideal contact pattern at the donor/acceptor interface to facilitate the smooth transfer of carriers to their respective electrodes. For obtaining high-performance PSCs with all-green solvent processing, revealing the morphological regulation process of PBHJ structure with green solvents is an urgent need.

In this work, we used the classical PM6 and BTP-eC9 donor–acceptor combination to fabricate the as-cast, BHJ, and PBHJ PSCs for systematically studying the effects of different active layer structures on the morphology regulation and device performance under green solvent processing. Compared with the as-cast film, the active layer morphology of BHJ film treated with DIO is significantly optimized. Consistent with previous research results, DIO can effectively control the morphology of BHJ films by delaying the drying time. Notably, the PBHJ structure enables the vertical phase separation of the donor–acceptor components in the active layer with p-i-n interaction, forming more direct and abundant carrier transport channels near the anode and cathode interfaces, respectively, thus having efficient carrier mobility. Thanks to the synergy effect of efficient exciton dissociation and balanced charge transport, the all-green processed PBHJ PSC has a champion PCE of 18.48%, and the short-circuit current density (*J*_sc_) is 27.45 mA cm^−2^, fill factor (FF) is 79.01%, and open-circuit voltage (*V*_oc_) is 0.852 V. The improvement of *J*_sc_ and FF is attributed to the good morphology of PBHJ films. This is one of the higher values for PSCs processed with green solvents. In short, the PBHJ structure PSCs combine the advantages of vertical component distribution in LbL and sufficient donor–acceptor interface in BHJ, providing a processing approach for efficient PSCs treated with a green solution.

## 2. Materials and Methods

**Materials:** Donor (PM6 and PTQ10), accepter (BTP-eC9), and the cathode buffer material (PDIN) were purchased from Solarmer Materials Inc. (Beijing, China). The PEDOT:PSS (Clevios P VP. AI 4083) was commercially procured from Xi’an Yuri Solar Co., Ltd. (Xi’an, China). The host solvent of *o*-xylene (*o*-XY) with over 99.9% purity, and the additive solvent of 1,8-diiodooctane (DIO) with over 99.8% purity, were procured from J&K Scientific Ltd. (Beijing, China) and Alfa Aesar Chemical Co., Ltd. (Shanghai, China), respectively. All materials were used without any further processing. All the tests were measured in an atmospheric environment without any encapsulation and room temperature.

**Methods:** The structure of the prepared devices were ITO/PEDOT:PSS/Active layers/PDIN/Ag. Firstly, the indium tin oxide (ITO) glass substrates with a sheet resistance of 10 Ω/□ and a size of 15 × 15 × 1.1 mm were continuously cleaned with special glass lotion, deionized water (resistivity ≥ 18 MΩ·cm) and ethanol in ultrasonic baths for 30 min in each step. Then, the ITO substrates were air-dried with high-purity nitrogen. To further improve the work function of the ITO substrates, the pre-cleaned ITO substrates were carried out with UV-ozone for 15 min. The aqueous solution of PEDOT:PSS was filtered through the 0.35 mm filter and then deposited on the pre-cleaned ITO substrate at 5000 rpm for 30 s. Before the substrate was transferred to the glove box, the PEDOT:PSS film was annealed at 150 °C for 15 min in air. For the as-cast device, the PM6 donor and BTP-eC9 acceptor were configured with *o*-XY mixed solution at 1:1.2 (*w*/*w*) with a total concentration of 15.4 mg mL^−1^ without the need for additives and post-processing. The BHJ device was fabricated by adding 0.4% (*v*/*v*) DIO into the *o*-XY mixed solution, and the mixture was spin-coated on the PEDOT:PSS at 2600 rpm with annealing at 90 °C for 5 min after spin-coating on the active layer. For the PBHJ device, the proportion conditions of active layers are shown in Supporting Information. After casting the first active layer at 2600 rpm for 25 s, the substrates with the film were transferred on a heating platform for 5 min, annealing at 90 °C to completely evaporate the solvent in it to form a strong film. In the same way, the second active layer was deposited. After that, the PDIN solution with a concentration of 2 mg mL^−1^ was spin-coated at a speed of 5000 rpm on the active layer for 30 s to form the cathode buffer layer. Finally, a roughly 100 nm-thick Ag electrode was deposited under vacuum conditions of 1 × 10^−4^ Pa. The active area of PSCs was about 0.04 cm^2^, and was named by the mask.

## 3. Results and Discussion

**Figure 1:**
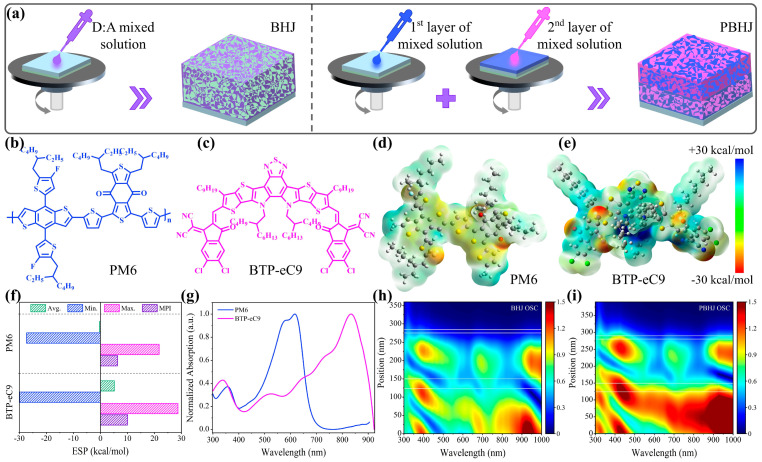
(**a**) Schematic diagram of the fabrication process for BHJ and PBHJ structural active layer films. Chemical structure of the active layer materials (**b**) PM6, (**c**) BTP-eC9 used in this work; The calculated ESP distributions of (**d**) PM6 and (**e**) BTP-eC9; (**f**) The values of molecular polarity index (MPI), maximal ESP (Max.), minimal ESP (Min.), and overall average (Avg.); (**g**) Normalized UV-vis absorption spectra of neat PM6 and BTP-eC9 film. Simulated optical field distribution of (**h**) BHJ PSCs and (**i**) PBHJ PSCs with the functional layers separated by the white solid lines.

First of all, Figure 1a describes the fabrication process for the traditional BHJ structure and the new PBHJ structure. The one-step spin-coating method is applied to deposit the donor:acceptor (D:A) blend solution in *o*-XY for BHJ films, while the PBHJ films are fabricated with different D:A ratios of diluted blend active layer solution in *o*-XY, respectively, and then spin-coated layer by layer. In this work, two representative star photovoltaic materials, PM6 and BTP-eC9 (we abbreviate it as eC9 in below), are taken as an example to study the difference in the influence of BHJ and PBHJ structures on device performance. The chemical structures of PM6 and BTP-eC9 are shown in Figure 1b,c. The density functional theory (DFT) with the B3LYP/6-31G (d, p) basis set is used to calculate the molecular frontier orbitals and electrostatic potential (ESP) distributions of these two materials as shown in Figure 1d,e. Details of the calculation process and the geometric optimization of the molecules and the frontier orbitals are shown in Supporting Information and Figures S1 and S2. The ESP distribution of BTP-eC9 has positive surface potential distribution on both the core skeleton and the inner and outer side chains, while the ESP distribution of PM6 shows more negative surface potential distribution. This indicates that appropriate intermolecular interactions can be formed between the donor and acceptor to promote the phase separation morphology [18,19]. Figure 1f further shows the extreme values and the overall average values of ESP distribution on the PM6 and BTP-eC9. Obviously, the average ESP of PM6 is negative, while that of BTP-eC9 is positive. Then, the molecular polarity index (MPI) is calculated based on the surface potential, and it is not difficult to see that PM6 and BTP-eC9 have similar MPI. This demonstrates that the two materials have a certain degree of compatibility and can form the blend phase [20,21]. The normalized absorption spectra of neat films with PM6 and BTP-eC9 are exhibited in Figure 1g, where they exhibit maximum absorptions at 617 nm and 833 nm, respectively, and complementary photon absorption. Prior to device preparation and characterization, we also simulated the optical field distribution for the BHJ and PBHJ PSCs with PM6:BTP-eC9 as seen in Figure 1h,i [22,23,24], and the variation of the extinction coefficient (k) and index of refraction (n) of the active layer as shown in Figure S3. In BHJ PSCs, the active layer thickness ranges from about 150–250 nm, while the active layer covers from 150 to 280 nm in PBHJ PSCs. The optical field intensity of the PBHJ active layer is obviously higher than that of BHJ, which means that the active layer of the PBHJ structure has higher light utilization. Meanwhile, the optical field distribution in the PEDOT:PSS and PDIN regions is also improved; that is, the PBHJ structure could increase the light utilization efficiency by decreasing the optical loss availably. Due to differences in the structure of the functional layer, the observed differences may result from the interference caused by the geometry of the layered device.

**Figure 2:**
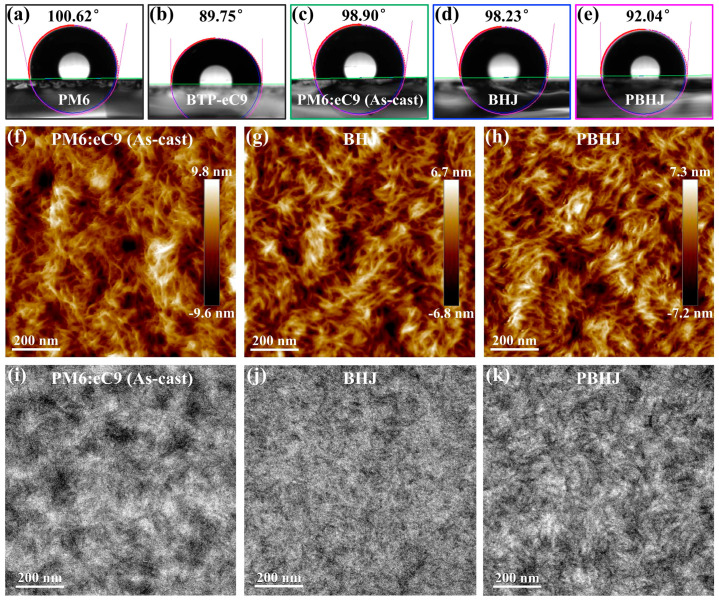
Contact angles of (**a**) PM6, (**b**) BTP-eC9 neat films and PM6:eC9’s (**c**) as-cast, (**d**) BHJ, (**e**) PBHJ films with water droplets. AFM height images of the (**f**) as-cast, (**g**) BHJ, (**h**) PBHJ films with PM6:eC9. TEM images of the (**i**) as-cast, (**j**) BHJ, (**k**) PBHJ films with PM6:eC9.

For elucidating the relationship between optical absorption and D-A migration and/or diffusion in BHJ and PBHJ structures, contact angle (CA) measurement with water and glycerol are carried out on the neat PM6 and BTP-eC9 films as well as the as-cast, BHJ, and PBHJ mixed films of PM6:eC9, respectively, as exhibited in Figure 2a–e and Figure S4. The surface phase component content of as-cast, BHJ, and PBHJ mixed films are calculated to employ the semi-quantitative method [25,26]. The water contact angles (WCA) of PM6 and BTP-eC9 neat films are 100.62° and 89.75°, respectively, while the WCA of as-cast, BHJ and PBHJ films with PM6:eC9 are 98.90°, 98.23° and 92.04°, respectively. The surface coverage fraction (*f*) of eC9 in three active layer films are calculated employing the Cassie–Baxter equation [27,28]: cos*θ* = *f* cos*θ*_eC9_ + (1 − *f*) cos*θ*_PM6_, where *θ* is the WCA value of the active layer films. The surface coverage of eC9 in as-cast, BHJ and PBHJ films are calculated to be 15.68%, 21.81% and 78.81%, respectively. These results showed that eC9 migrated to the upper surface of the blended film along the volatilization direction due to small convection induced by volatilization of solvents and additives, which is beneficial to the formation of vertical gradient donor–acceptor BHJ phase structure [28]. In the PBHJ structure, eC9 occupied the dominant proportion in the second mixed solution, and the higher concentration distribution made its enrichment on the upper surface more significant, which is conducive to increasing charge transfer and thus improving PSC performance. Furthermore, the surface tension is calculated according to the glycerol CA of PM6 and BTP-eC9 neat films and as-cast, BHJ, and PBHJ films with PM6:eC9 are 18.90, 24.49, 19.85, 19.92, 23.61 mN m^−1^ (Table S1), respectively. It is not difficult to see that the surface tension of PBHJ film (23.61 mN m^−1^) is nearer to that of neat eC9 film (24.49 mN m^−1^), indicating that eC9 is more inclined to gather at the upper interface, which is in line with the WCA analysis results. To clearly verify the morphological changes in different active layers, we conducted the striking atomic force microscopy (AFM) characterization. The height images and corresponding phase images are placed in Figure 2f–h and Figure S5, respectively. The root mean square roughness (R_q_) of the as-cast film with PM6:eC9 is 2.76 nm, and after treating with the DIO additive, the R_q_ of BHJ film is reduced to 1.89 nm, which indicates that the surface of BHJ film is more uniform and smooth, and the phase separation is smaller. For PBHJ films, the R_q_ value is slightly increased to 2.08 nm compared with BHJ films, and the surface aggregation of the acceptor phase can be obviously observed, which may be the result of enhanced crystallinity in the PBHJ structure [29,30]. In the AFM phase images, the phase separation within the three active layer films is also obvious. In addition, from the transmission electron microscopy (TEM) image in Figure 2i–k, the PM6:eC9 as-cast film has a large number of white and black patches, which leads to excessive phase separation. BHJ films exhibit a flocculation phenomenon with no obvious regional differences, which limits the sufficient contact between the donor and acceptor and insufficient exciton dissociation. It is worth mentioning that the degree of phase separation in PBHJ films is significantly improved, showing a continuous interpenetrating network configuration with some tiny eC9 crystals (black), which may provide a more convenient transport pathway for the carriers [31]. The observed results of CA are consistent with the surface results of AFM and TEM. More acceptor molecules are gathered on the upper surface of the active layer in the PBHJ structure to form more favorable charge transfer channels.

**Figure 3:**
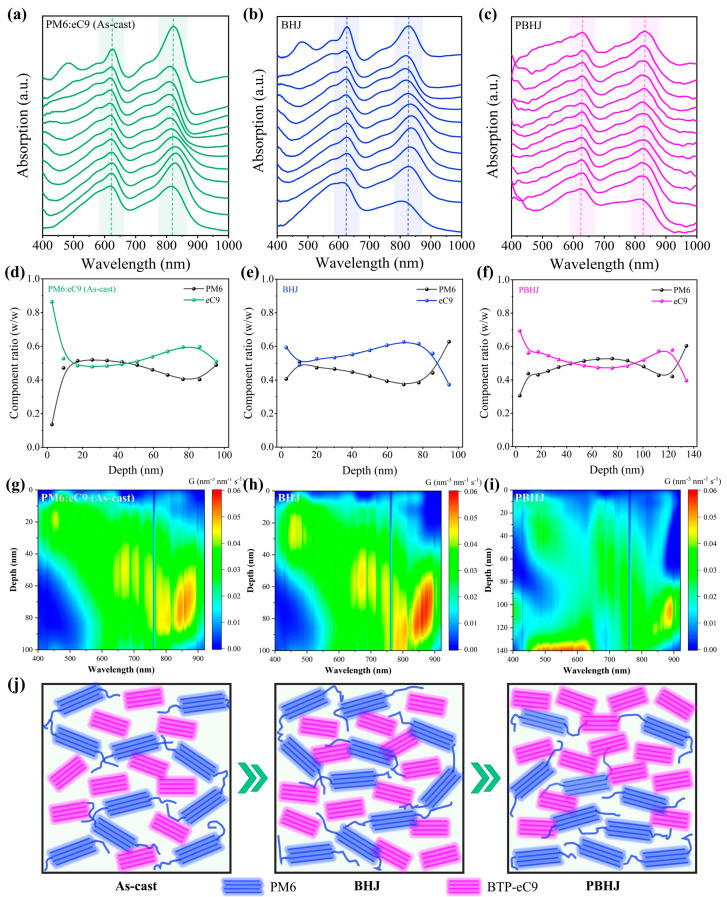
Film-depth-dependent light absorption spectra of (**a**) as-cast, (**b**) BHJ, (**c**) PBHJ with PM6:eC9 system mixed films. (**d**–**f**) The calculated composition distribution of films with different active layers at different film depths. The simulated exciton generation contours in (**g**) as-cast, (**h**) BHJ, (**i**) PBHJ films. (**j**) Schematic diagram of morphological evolution of the as-cast, BHJ, PBHJ films.

To better understand the effects of different active layers on the vertical distribution on PM6 and BTP-eC9, film-depth-dependent light absorption spectroscopy (FLAS) is conducted. The sublayer absorption spectroscopy and in-situ etching spectrum of as-cast, BHJ, and PBHJ with PM6:eC9 film can be seen in Figure 3a–c and Figure S6 [32,33]. The three kinds of mixed films show hierarchical vertical phase segregation with a small degree of energetic disorder. The absorption peak intensities of donor and acceptor in BHJ and PBHJ films are more balanced, while the absorption peak intensity of PM6 donor gradually decreased, and the absorption peak intensity of eC9 acceptor gradually increased in the PBHJ film, which confirmed the vertical phase distribution of PBHJ [34]. Further, we calculated the component distribution under different film depths corresponding to the three mixed films as shown in Figure 3d–f. The bottom (~100 nm) of BHJ and PBHJ films are rich in PM6 donors, which proves the existence of favorable vertical phase separation. It is worth noting that the top of the PBHJ film is obviously enriched with more eC9 acceptors, and the flatter component ratio curve indicates that the blending region is larger, which is conducive to the formation of the p-i-n structure. Then, the exciton generation contour and rate in the active layer are simulated according to the improved transfer-matrix optical model, as seen in Figure 3g–i and Figure S7. It is not difficult to see that BHJ has a higher exciton generation rate from 60 to 100 mn of the depth range compared with the as-cast active layer, and the position of the maximum exciton production rate is about 53 nm, which has a more moderate position to reduce charge recombination caused by long-distance transport compared to as-cast film (43 nm) [35,36]. The contour of exciton generation rate in PBHJ film is more uniform, and two peaks appear at about 47 nm and 120 nm, respectively, which reveals that the PBHJ structure is conducive to rapid dissociation of exciton generation to electrode transport, resulting in high short-circuit current generation. This is discussed further in the device testing section below.

**Figure 4:**
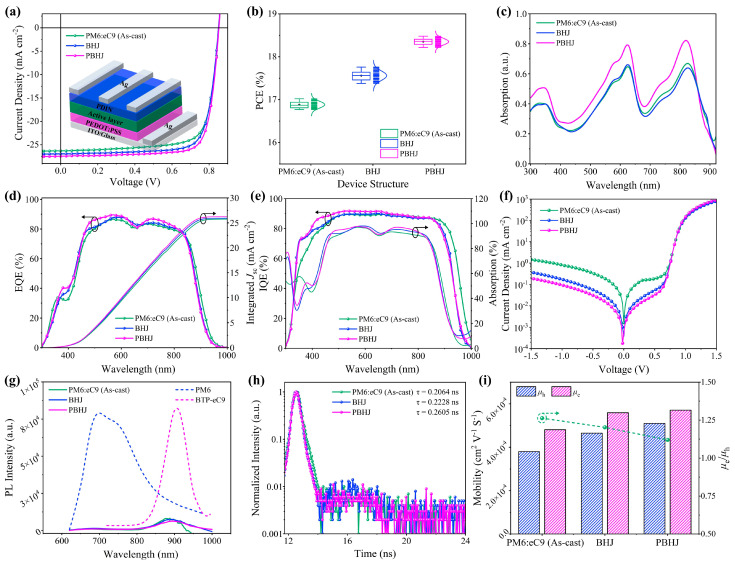
(**a**) *J*-*V* characteristics of the as-cast, PM6:eC9 system BHJ and PBHJ devices; (**b**) PCE measurement statistics from 30 devices; (**c**) Normalized UV-vis absorption spectra of PM6:eC9 films with as-cast, BHJ and PBHJ; (**d**) EQE curves and integral *J*_sc_; (**e**) IQE curves and absorption spectra; (**f**) Dark current curves of the PM6:eC9 devices with as-cast, BHJ and PBHJ; (**g**) PL spectra of BTP-eC9 and PM6 neat films and as-cast, BHJ, PBHJ mixed films based on PM6:eC9 system; (**h**) Normalized TRPL decay curves for PM6:eC9 as-cast, BHJ and PBHJ films; (**i**) Hole and electron mobility measured in the as-cast, BHJ and PBHJ films with the PM6:eC9 system in single-carrier diodes.

In our work, the conventional device structure with indium tin oxide (ITO)/poly(3,4-ethylenedioxythiophene): poly(styrene sulfonate) (PEDOT:PSS)/active layers/poly(fluorene) derivatives and perylene diimide (PDIN)/Ag were used to fabricate the PSCs for studying the influences of the BHJ and PBHJ structures on device performance. Detailed information on the fabricating processes of the device can be found in Supporting Information. The current density–voltage (*J*-*V*) characteristic curves and corresponding photovoltaic parameters of as-cast, BHJ, and PBHJ optimal devices with PM6:eC9 system are shown in Figure 4a and Table 1. In addition, the photovoltaic performance of PBHJ structured devices with different D:A proportional dilution is investigated in Table S2. The PCE of the as-cast device with PM6:eC9 is 17.02%, short-circuit current density (*J*_sc_) is 26.30 mA cm^−2^, open-circuit voltage (*V*_oc_) is 0.853 V, and fill factor (FF) is 75.85%. After DIO treatment, the PCE of BHJ devices enhanced to 17.76%, the FF raised to 77.48%, *J*_sc_ increased to 26.93 mA cm^−2^, and *V*_oc_ decreased to 0.851 V. In general, the *V*_oc_ of BHJ devices after DIO treatment will be slightly reduced, and this is similar to previously reported research [37,38]. For PBHJ PSCs, the FF and *J*_sc_ are further improved to 79.01% and 27.45 mA cm^−2^, and *V*_oc_ is slightly improved to 0.852 V compared to BHJ devices, thus achieving a champion PCE of 18.48%. This is comparable in PSCs treated with green solvent (Table S3). For determining the reliability and stability of device performance changes under different structures, the PCE distribution statistics are provided using 30 device performances for each system, as can be seen in Figure 4b. Obviously, PBHJ structure PSC photovoltaic performance is better than BHJ structure and PCE has high repeatability. This is also well supported in the PTQ10:eC9 system (Table 1 and Figure S8). We found that the significant enhancement in FF and *J*_sc_ is mainly responsible for the improved performance of the PBHJ PSCs. The improvement in FF comes from optimizing the morphology of the active layer [39,40]. The optical field distribution of PBHJ PSCs simulated in Figure 1i shows that the PBHJ structure can improve the optical utilization efficiency by effectively reducing the optical loss. Therefore, as shown in Figure 4c, the normalized UV-vis absorption spectra of the active layer are examined. The results show that the absorption intensity of PBHJ film is significantly higher than that of the BHJ and as-cast films, and the absorption peak is almost unchanged. Then, the external quantum efficiencies (EQE) and integrated *J*_sc_ are used to illustrate the increased trend of the *J*_sc_, as seen in Figure 4d. There are reasonable differences between the *J*_sc_ values calculated by the EQE curve and the *J*_sc_ values tested by the corresponding *J*-*V* curve. The EQE response of PBHJ devices at 400–850 nm is dramatically higher than that of BHJ PSCs, which is consistent with its absorption test and high *J*_sc_ value. Figure 4e displays the internal quantum efficiencies (IQE) and the absorption spectra of the corresponding devices. PBHJ devices have larger IQE values over the entire wavelength range, suggesting that the increased *J*_sc_ may primarily benefit from efficient charge separation, transport, and collection, which is discussed further below [39]. Moreover, the shunt resistance (R_sh_) of the PBHJ structure solar cells is higher than that of the BHJ and as-cast structure solar cells, resulting in a lower reverse saturated current density; that is, the leakage current from the device is suppressed. The series resistance (R_s_) of the PBHJ device is also further reduced, resulting in a higher FF. Larger R_sh_ and smaller R_s_ favor charge transport and collection, which is consistent with IQE results [30]. The dark *J*-*V* characteristic curve in Figure 4f shows that the current density of PBHJ devices in the negative bias region is significantly lower than that of the BHJ and as-cast devices. This indicates that PBHJ PSCs have a higher rectification ratio and better diode characteristics, which effectively decreases the loss of internal charge and leakage current. This improves the device of the *J*_sc_ and FF [41,42].

Further, the steady-state photoluminescence (PL) measurements are performed on PM6 and BTP-eC9 neat films and mixed films with PM6:eC9 systems under excitation of 600 nm in Figure 4g. The PL spectra of eC9 neat film have a strong photoluminescence peak at 907 nm, but the PL intensity of all mixed films can be ignored. PBHJ film even exhibits more efficient quenching and lower PL intensity, which indicates that PBHJ film has sufficient D:A interfacial area for charge transfer [43,44,45]. The transient photoluminescence (TRPL) is applied to test the exciton lifetime (*τ*) in the mixed films as shown in Figure 4h. It is easy to see that the as-cast, BHJ, and PBHJ mixed films with PM6:eC9 system have similar exciton lifetimes because the used material of the active layer is the same. However, the average exciton lifetime of the PBHJ film is longer (*τ* = 0.2605 ns). In addition, based on the Mott–Gurney equation, the space-charge limited current (SCLC) method is applied to calculate the charge mobility [45]. We carried out the single carrier devices of ITO/PEDOT:PSS/Active layers/MoOx/Ag and ITO/ZnO/Active layers/PDIN/Ag to measure the charge mobility of electron and hole, respectively. The *J*^1/2^-*V* characteristics of the devices are exhibited in Figure S9 and the values of electron mobility (*µ*_e_) and hole mobility (*μ*_h_) are displayed in Figure 4i and Table S4, respectively. For PM6:eC9 as-cast device, the *μ*_h_, *µ*_e_ and *μ*_h_/*µ*_e_ are 3.80 × 10^−4^, 4.81 × 10^−4^ cm^2^ V^−1^ s^−1^ and 1.263, respectively. The *μ*_h_ and *µ*_e_ are increased to 4.65 × 10^−4^ and 5.59 × 10^−4^ cm^2^ V^−1^ s^−1^ and *μ*_h_/*µ*_e_ is 1.202 in PM6:eC9 BHJ device, indicating a more balanced charge transfer in BHJ device. For the PBHJ structure, the values of *μ*_h_ and *µ*_e_ increase significantly to 5.10 × 10^−4^ and 5.71 × 10^−4^ cm^2^ V^−1^ s^−1^, and the value of *μ*_h_/*µ*_e_ (1.120) is closest to 1, indicating that the best-balanced charge transport path is formed in the PBHJ device. In other words, the increase in *J*_sc_ and FF benefits from more balanced carrier mobility performance.

**Figure 5:**
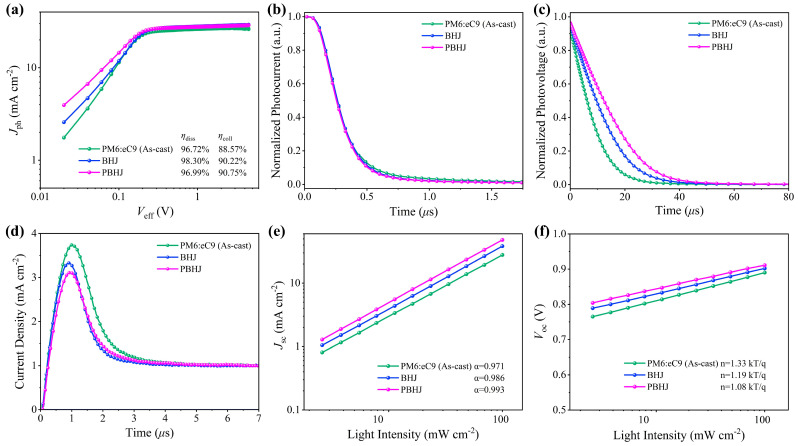
(**a**) Photocurrent versus effective voltage, (**b**) TPC curves, (**c**) TPV curves, (**d**) Photo-CELIV, (**e**) *J*_sc_ versus light intensity (*P*_light_) data and power-law (*J*_sc_ ∝ *P*_light_^α^) fit, (**f**) *V*_oc_ versus *P*_light_ data and power-law (*V*_oc_∝n*kT*/*q*log*P*_light_) fit of the as-cast, BHJ and PBHJ devices based on PM6:eC9 system.

For illustrating how charge generation, extraction, and recombination characteristics of different active layers improve the photovoltaic performance in PSCs, the relationship between the effective voltage (*V*_eff_) and the photocurrent density (*J*_ph_) of the devices are recorded, as seen in Figure 5a. PBHJ devices are significantly more likely to reach the *J*_ph_ saturation state (*J*_sat_), indicating a higher efficiency in charge generation and collection. In addition, the *J*_ph_ values under short circuit and maximum power output conditions are *J*_ph_^*^ and *J*_ph_^∞^, respectively, which can be further calculated exciton dissociation probability (*η*_diss_) and charge collection efficiency (*η*_coll_) of the films [46,47]. Details of the *J*_ph_, *J*_sat_ and their ratios are shown in Table S5. The *η*_diss_ of as-cast, BHJ, and PBHJ devices with PM6:eC9 system are 96.72%, 98.30% and 96.99%, respectively, while the *η*_coll_ in different devices are 88.57%, 90.22% and 90.75%, respectively. It is obvious that PBHJ devices have more efficient exciton dissociation and charge collection. Moreover, the transient photocurrent (TPC) and transient photovoltage (TPV) are effective tools for the quantitative analysis of charge lifetime and charge extraction capability in PSCs, as seen in Figure 5b,c [48,49]. The charge extraction time (*τ*_ext_) is estimated by fitting the TPC decay curves, and the smallest *τ*_ext_ from PBHJ devices is 0.23 μs, indicating that the vertical distribution in PBHJ devices effectively promotes the carrier extraction. This is consistent with the above-mentioned results for high charge mobility and charge collection efficiency. The photocarrier lifetime (*τ*_pho_) under open circuit condition is estimated via fitting the TPV decay curves, and the longest *τ*_pho_ of PBHJ device is 16.65 μs, obviously due to the 13.17 μs in the *BHJ* device. The results indicate that the charge recombination is the weakest in the PBHJ device, which is consistent with the above TRPL result. The photoinduced charge extraction in linearly increasing voltage (Photo-CELIV) is used for estimating the *μ* value of the devices, as shown in Figure 5d and Table S6. The optimal *μ* value of 2.00 × 10^−4^ cm^2^ V^−1^ s^−1^ supports the most efficient charge extraction in PBHJ device, compared to 1.66 × 10^−4^ cm^2^ V^−1^ s^−1^ for the as-cast device with PM6:eC9 and 1.89 × 10^−4^ cm^2^ V^−1^ s^−1^ for BHJ device. In addition, the light intensity (*P*_light_) dependence of the *J*_sc_ reflects the degree of exciton recombination in the device, as shown in Figure 5e [50]. The corresponding relationship between *J*_sc_ and *P*_light_ is *J*_sc_ ∝ *P*_light_^α^, where *α* is the exponential factor of bimolecular recombination, and *α* approaching 1 indicates that bimolecular recombination is inhibited. The *α* values of as-cast, BHJ, and PBHJ devices for PM6:eC9 fitted are 0.971, 0.986 and 0.993, respectively, indicating that the loss of PBHJ PSCs is minimal through bimolecular recombination. The dependence of the *V*_oc_ on the *P*_light_ is usually *V*_oc_ ∝ n*kT*/*q*log*P*_light_, as shown in Figure 5f, where *k* is the Boltzmann constant, n is the constant, *q* is the elementary charge, and *T* is the Kelvin temperature [51,52,53]. When the n approaches 1, trap-assisted or monomolecular recombination is effectively inhibited in the device. The n values of the as-cast, BHJ and PBHJ devices with PM6:eC9 fitted are 1.33, 1.19 and 1.08, respectively, indicating that the PBHJ devices exhibit the smallest trap-assisted recombination. In a word, proper vertical distribution and phase separation in PBHJ PSCs play key roles in inhibiting charge recombination and promoting efficient charge generation and collection.

## 4. Conclusions

In summary, we fabricated a PBHJ structure PM6:BTP-eC9-based polymer solar cell with cross-distribution in the vertical direction via preparing two layers of the *o*-XY solution with different dilution ratios of donor–acceptor mixes. The morphological differences between the PBHJ structure and that of BHJ and as-cast films were systematically studied. The PBHJ film has suitable nanoscale interpenetrating network morphology and forms vertical phase separation of donor and/or acceptor layer interaction p-i-n structure on the vertical scale to optimize carrier transport pathways. Charge dynamics measurements also show that the PBHJ devices with good vertical distribution are conducive to exciton dissociation and charge transfer, which results in the charge mobility being more balanced, and the FF and *J*_sc_ of the devices become significantly improved. Finally, the binary PBHJ PSCs based on PM6:BTP-eC9 achieve a *V*_oc_ of 0.852 V, *J*_sc_ of 27.45 mA cm^−2^, and FF of 79.01%, and thus have a champion PCE of 18.48% under green processing. Moreover, the PBHJ structure is well verified in the PTQ10:BTP-eC9 system. Our results provide an all-green solution treatment approach for morphological regulation to guide the targeted optimization of device performance.

## Figures and Tables

**Table 1:** The photovoltaic parameters with different structural PSCs.

Systems	Treatment Strategy	*V*_oc_ (V)	*J*_sc_ (mA cm^−2^)	FF (%)	PCE (%) Max. ^a^	PCE (%) Avg. ^b^	R_s_ (Ω cm^−2^)	R_sh_ (Ω cm^−2^)
PM6:BTP-eC9	As-cast	0.853	26.30	75.85	17.02	16.90 ± 0.13	95.26	16,746.24
	BHJ	0.851	26.93	77.48	17.76	17.57 ± 0.18	83.55	22,482.77
	PBHJ	0.852	27.45	79.01	18.48	18.35 ± 0.13	74.68	29,320.11
PTQ10:BTP-eC9	BHJ	0.883	24.75	72.34	15.81	15.66 ± 0.15	101.56	18,478.11
	PBHJ	0.885	25.56	74.39	16.83	16.72 ± 0.11	98.17	20,853.32

^a^ The maximum value comes from the most efficient solar cell device; ^b^ The average values are derived from 30 identical batches of solar cell devices.

## Data Availability

Data are contained within the article and Supplementary Materials.

## Supplementary Materials

The following supporting information can be downloaded at: https://www.mdpi.com/article/10.3390/polym17030284/s1, Figure S1: The front-view and side-view of PM6 and BTP-eC9 molecular geometry optimized via the density functional theory of B3LYP/6-31G (d, p) basis set; Figure S2: The HOMO and LUMO energy level contributions of (a, b) PM6 and (c, d) BTP-eC9 are calculated by DFT. (e) Schematic comparison of the energy levels from DFT with the reported in the literature of PM6 and BTP-eC9; Figure S3: The curves of index-n and -k variation with wavelength of the (a) BHJ and (b) PBHJ active layer; Figure S4: Contact angles of (a) PM6, (b) BTP-eC9 neat films and PM6:eC9’s (c) as-cast, (d) BHJ, (e) PBHJ films with glycerin droplets; Figure S5: AFM phase images of the (a) as-cast, (b) BHJ, (c) PBHJ films with PM6:eC9; Figure S6: Film-depth-dependent light absorption in situ etching spectra of (a) as-cast, (b) BHJ, (c) PBHJ with PM6:eC9 system mixed films; Figure S7: The simulated the film-depth-dependent exciton generation rates in (a) as-cast, (b) BHJ, (c) PBHJ films; Figure S8: (a) J-V characteristics of the BHJ and PBHJ devices based on the PTQ10:eC9 system, (b) PCE measurement statistics from 30 devices; Figure S9: SCLC characteristics of the (a) hole-only, and (b) electron-only of as-cast, BHJ, and PBHJ devices; Table S1: The surface tension information of PM6, BTP-eC9 neat film and PM6:eC9 mixed film from contact angle; Table S2: J-V characteristics of PM6:eC9 PBHJ devices with different dilution ratios; Table S3: The photovoltaic performance of binary PSCs reported in the literature; Table S4: Hole and electron only mobility of devices with different active layer structures; Table S5: The key parameters of the photocurrent versus effective voltage with different active layer devices based on PM6:eC9 system; Table S6: The key parameters of *τ*_ext_, *τ*_pho_, and *μ* for the devices with the different active layer based on PM6:eC9 system. References [12,14,17,32,33,35,40,54,55,56,57,58,59,60,61,62,63,64,65] are cited in the Supplementary Materials.
