# Supplementary material for: The Pseudo-Bilayer Bulk Heterojunction Active Layer of Polymer Solar Cells in Green Solvent with 18.48% Efficiency
Source: Polymers (Basel). 2025 Jan 22;17(3):284. doi: 10.3390/polym17030284 (PMC11819932; doi:10.3390/polym17030284)
Supplement: Supplementary file 1 [file polymers-17-00284-s001.zip › polymers-3423712-supplementary.pdf]

Supplementary Information

The pseudo-bilayer bulk heterojunction active layer of organic solar cells in green solvent with 18.48% efficiency

Jingyue Cao[1,2], Zheng Xu[1,2 *]

[1,] Key Laboratory of Luminescence and Optical Information, Beijing Jiaotong University, Ministry of Education, Beijing 100044, China.

[2,] Institute of Optoelectronics Technology, Beijing Jiaotong University, Beijing 100044, China.

[*]Corresponding author: zhengxu@bjtu.edu.cn

Experimental details:

Device characterization:

The current–voltage (*J-V*) characteristics of the devices were measured with a Keithley 2400 semiconductor characterization system under the simulated AM 1.5G spectrum with a power of 100 mW cm^{-2} generated by a 150 W solar simulator with AM 1.5G filters (PV Measurements), which was calibrated by standard silicon solar cells (purchased from Enlitech). An external quantum efficiency (EQE) spectrum was analyzed by using a PV Measurement QEX7 system. The electron and hole mobility were measured by using the method of space-charge limited current (SCLC) for electron-only device with the structure of ITO/ZnO/Active layers/PDIN/Ag and hole-only device with the structure of ITO/PEDOT:PSS/Active layers/MoO_x/Ag. The charge carrier mobility was determined by fitting the dark current to the model of a single carrier SCLC according to Equation (S1, S2):

$$J = \frac{9\varepsilon_0\varepsilon_r\mu V^2}{8d^3} \quad \text{(S1)}$$

$$V = V_{appl} - V_{bi} - V_s \quad \text{(S2)}$$

where J is the current density, ε_0 is the permittivity of free space, ε_r is the relative dielectric constant of the transport medium, μ is the charge carrier mobility, and d is the film thickness of the active layer. Within this, V is obtained by Equation (2), where V_{appl} is the applied voltage, V_{bi} is the offset voltage, and V_s is the voltage drop due to contact resistance and series resistance across the electrodes. The carrier mobility can be calculated from the slope of the $J^{1/2}$~V curves.

Transient photovoltage (TPV), transient photocurrent (TPC), and photo-induced charge extraction linear increasing voltage (Photo-CELIV) were conducted with the Paioscarrier measurement system (FLUXiM AG, Switzerland). A high-power white LED was utilized as the light source for TPV, TPC, and photo-CELIV measurements. The integrated power of the LED was 72 mW cm^{-2}, and the spectrum distribution was mainly in the wavelength range of 440-470 nm and 540-630 nm, and the peak value was located at 460 nm and 550 nm.

Morphology characterizations:

The absorption spectra of thin films were recorded on a Shimadzu UV-3101 PC spectrophotometer. The photoluminescence (PL) spectra were obtained using a femtosecond broadband supercontinuum probe pulse that was overlapped in time and space with the femtosecond pump pulse. The supercontinuum was produced by focusing a small portion of the amplified laser fundamental into a sapphire plate. Multi-wavelength transient spectra were recorded using dual spectrometers (signal and reference) equipped with fast Si array detectors. The fluence value was fixed at 16 μJ cm^{-2} after determining the beam spot size. Dispersion in the white probe beam was corrected before the data analysis. The morphology of the thin films was investigated through atomic force microscopy (AFM) Bruker Dimension ICON operated in fast scan mode with reflective probes resonating at 150 kHz frequency, and all the samples were measured with a scan size of 2*2 μm^2. Transmission electron microscopy (TEM) images of the active layers were

obtained by using a JEOL JEM-1400 transmission electron microscope operated at 80 kV. The samples for AFM and TEM characterization were prepared under the same conditions compared with the active layers of the OSCs. The samples for TEM measurement were prepared by dissolving the PEDOT:PSS layer using deionized water and picking up the active layer using 400-mesh copper TEM grids. The contact angle measurements were performed by Rame–Hart goniometer in sessile drop mode. The interfacial surface energy values of materials can be obtained directly by instrument, and also according to Young's equation.

The film-depth-dependent light absorption spectroscopy was acquired from a film-depth-dependent light absorption spectrometer (PU100, Puguangweishi Co. Ltd). In-situ soft plasma etching at low pressure (less than 20 Pa) was used to extract the depth-resolved absorption spectrum for the organic active layer. From the evolution of the spectra and Beer–Lambert's Law, film-depth-dependent absorption spectra were extracted based on the structure of ITO/PEDOT:PSS/active layer. The probed depth of the active layer was approximately 100 nm, which corresponds to the thickness of the blend films. The composition distribution along the film-depth direction was obtained from the film-depth-dependent spectra. The detailed experimental and numerical methods can be found in the literature [1-3].

Computational Methods:

The geometries of PM6 and BTP-eC9 were all optimized with dispersion-corrected density functional theory (DFT-D3) at the B3LYP-D3/6-31G(d) level. [4-6] In order to describe the solvation effect, the Solvation Model Based on Density (SMD) implicit solvent model was used in all calculations [7]. To obtain the electron energy with higher accuracy, single point calculations for these optimized structures with B3LYP functional and 6-311G(d,p) basis set were performed. The electrostatic surface potential (ESP) and its distribution of these three molecules were calculated using the Multiwfn program and then rendered using the GaussView program [8, 9]. The vibrational frequencies were

calculated after geometry optimization and no imaginary frequency was found. All the calculations were performed in the Gaussian 16 package.

In this work, we calculated the optical field distribution of the nanostructures using FDTD. During the computation, we set the *x* and *y* directions as periodic boundary conditions and the *z* direction as a perfectly matched layer condition. In addition, for obtaining the accurate calculation results, we divided the mesh of the whole simulation area into 2nm × 2nm × 2nm. Importantly, we employed a plane wave as an excitation light source incident vertically along the *z*-axis onto the surface of the nanostructures. We confirmed the convergence time as 1×10^{-5} *f*s to ensure the convergence of the computational results. Finally, an electric field monitor was employed to gain the electric field distribution of the nanostructures.

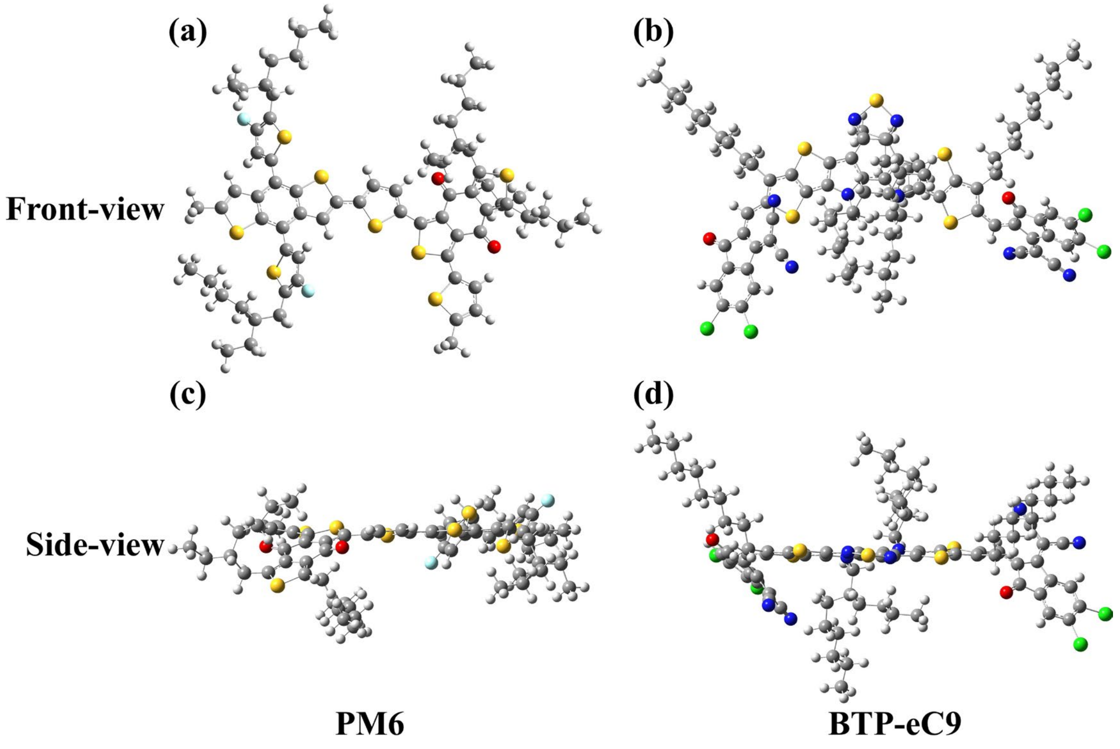

Figure S1 The front-view and side-view of PM6 and BTP-eC9 molecular geometry optimized via the density functional theory of B3LYP/6-31G (d, p) basis set.

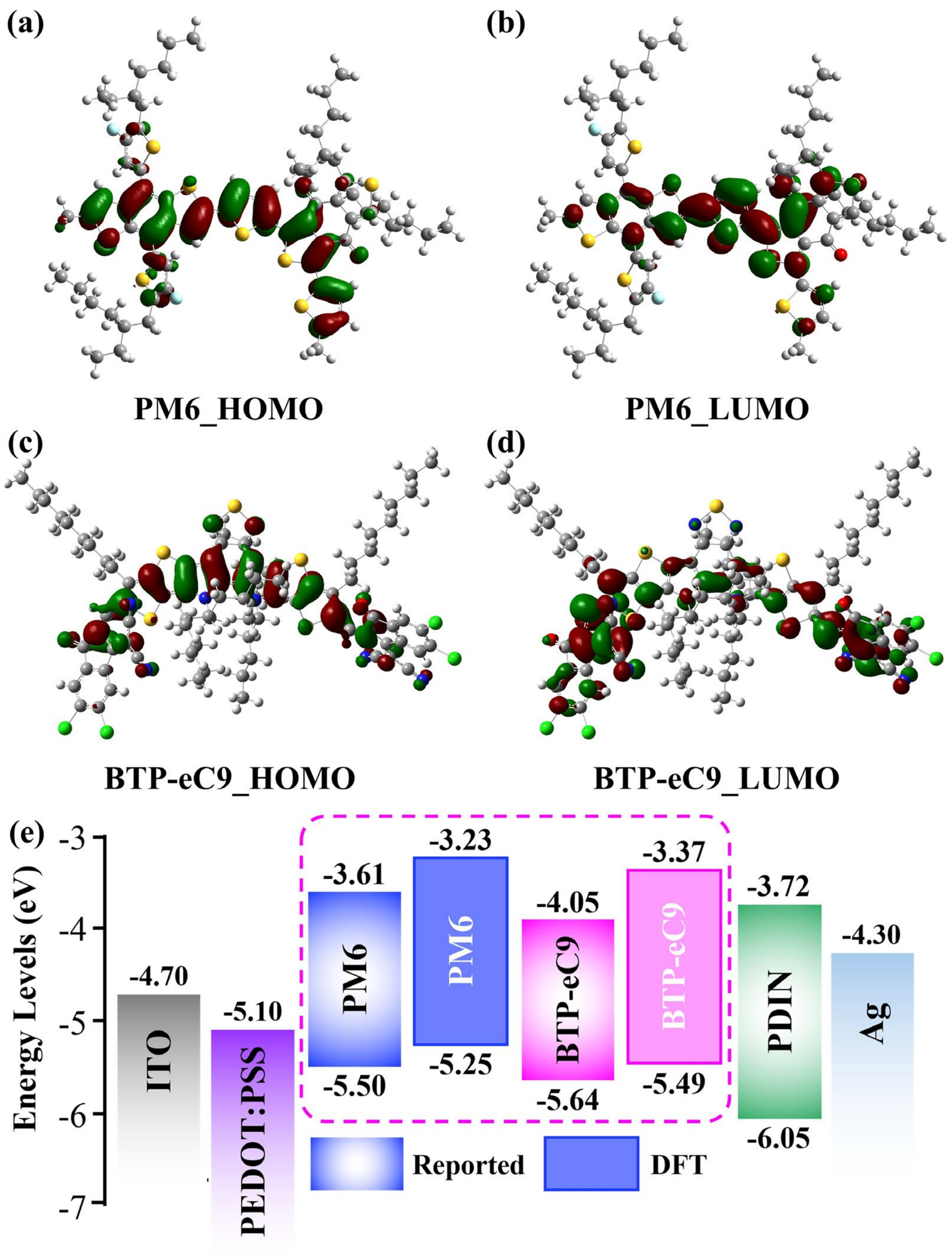

Figure S2 The HOMO and LUMO energy level contributions of (a, b) PM6 and (c, d) BTP-eC9 are calculated by DFT. (e) Schematic comparison of the energy levels from DFT with the reported in the literature of PM6 and BTP-eC9 [10, 11].

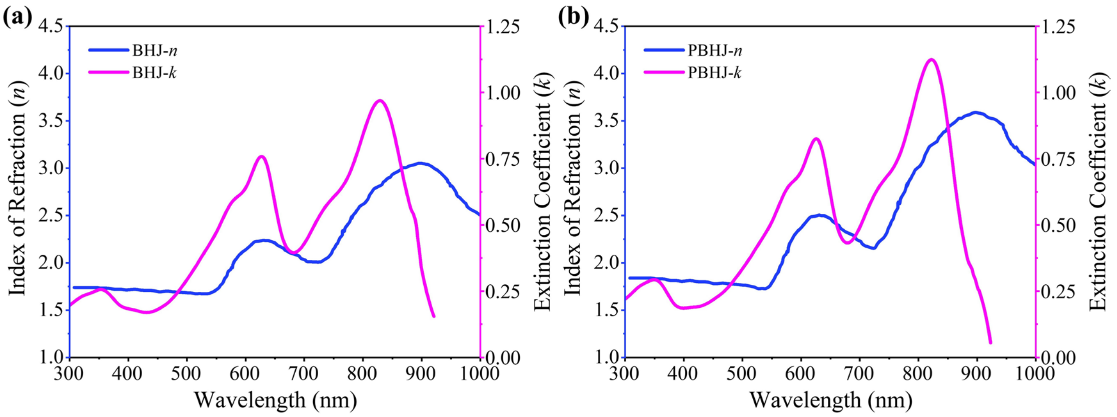

Figure S3 The curves of index-*n* and -*k* variation with wavelength of the (a) BHJ and (b) PBHJ active layer.

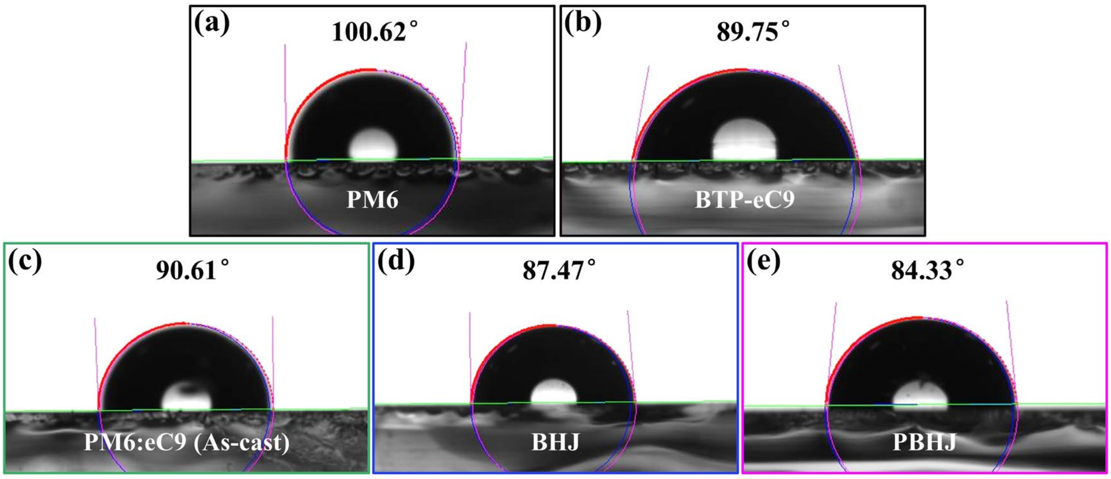

Figure S4 Contact angles of (a) PM6, (b) BTP-eC9 neat films and PM6:eC9's (c) as-cast, (d) BHJ, and (e) PBHJ films with glycerin droplets.

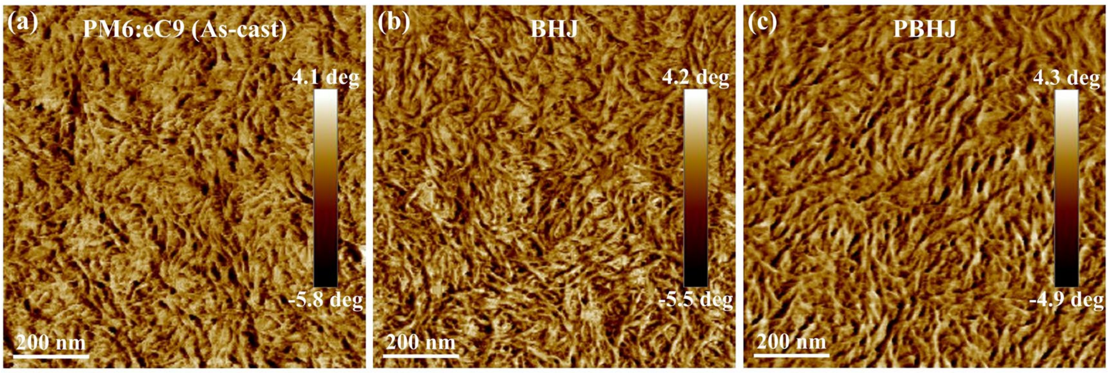

Figure S5 AFM phase images of the (a) as-cast, (b) BHJ, and (c) PBHJ films with PM6:eC9.

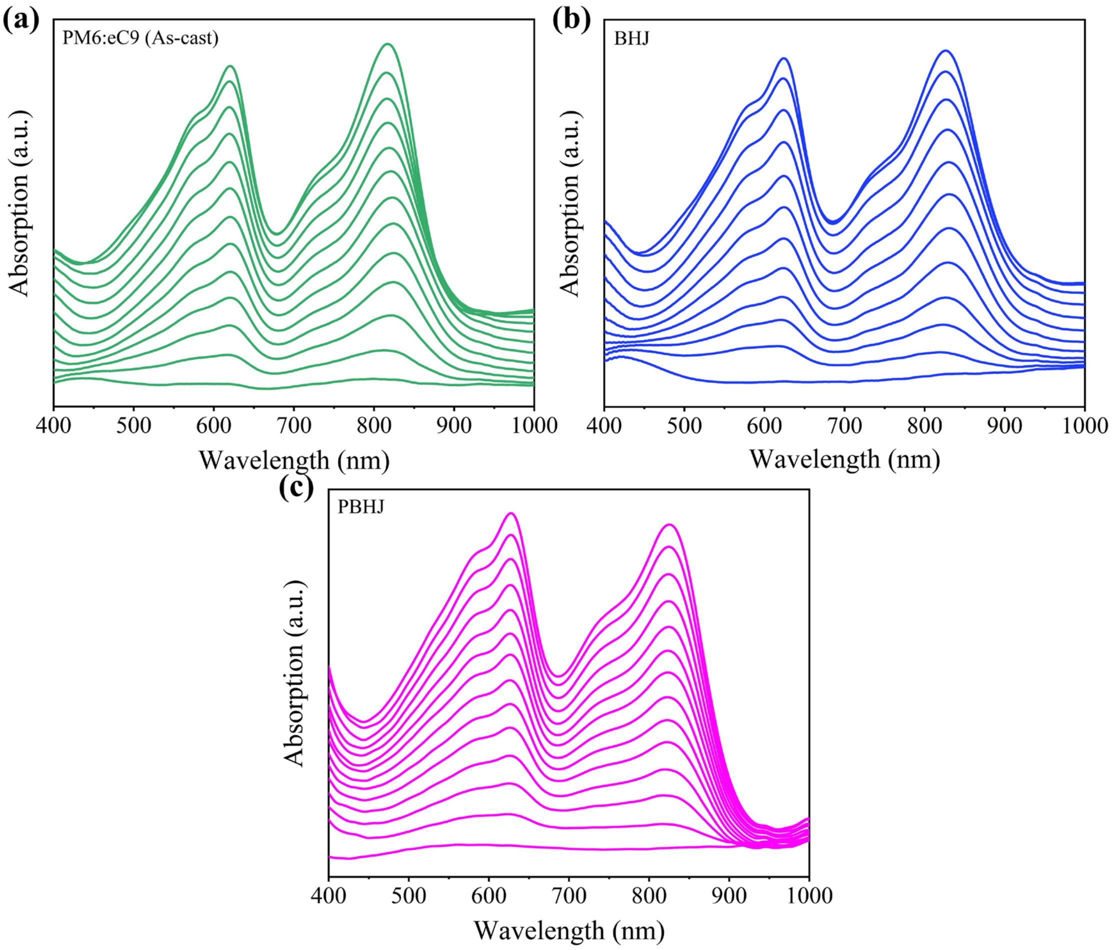

Figure S6 Film-depth-dependent light absorption in situ etching spectra of (a) as-cast, (b) BHJ, and (c) PBHJ with PM6:eC9 system mixed films.

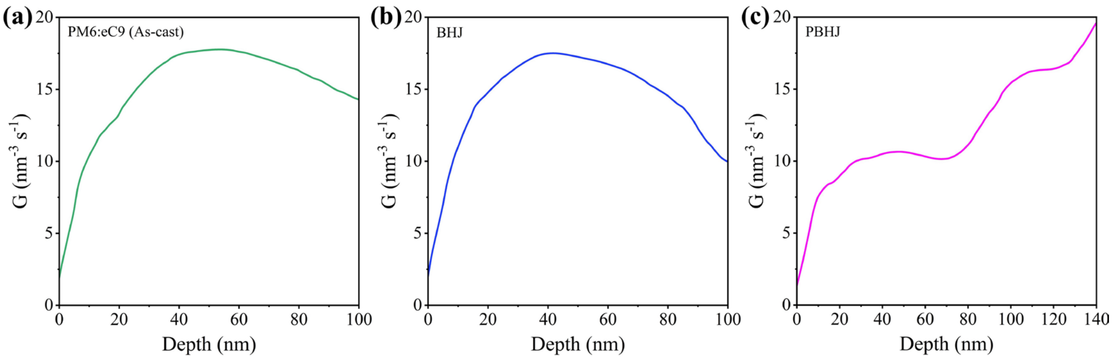

Figure S7 The simulated film-depth-dependent exciton generation rates in (a) as-cast, (b) BHJ, and (c) PBHJ films.

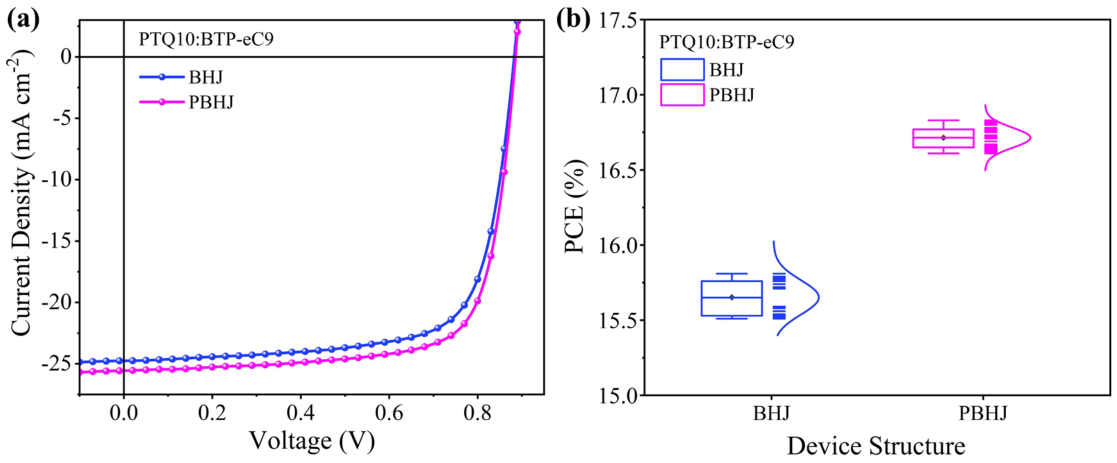

Figure S8 (a) *J-V* characteristics of the BHJ and PBHJ devices based on the PTQ10:eC9 system and (b) PCE measurement statistics from 30 devices.

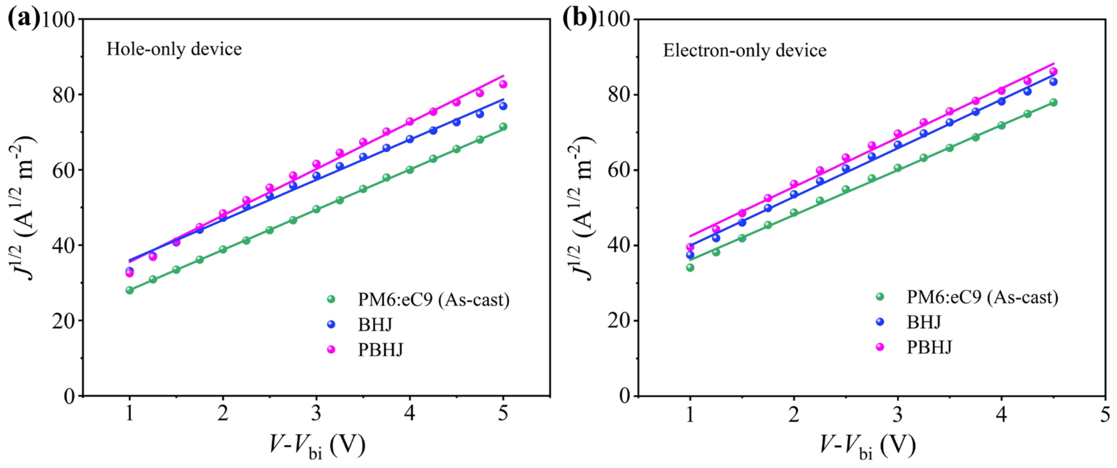

Figure S9 SCLC characteristics of the (a) hole-only, and (b) electron-only as-cast, BHJ, and PBHJ devices.

Table S1 The surface tension information of PM6, BTP-eC9 neat film and PM6:eC9 mixed film from contact angle.

Systems	θ_{water} (º)	$\theta_{glycerol}$ (º)	γ_d (mN m^{-1})	γ_p (mN m^{-1})	γ_{tot} (mN m^{-1})
PM6	100.62	91.76	3.504	15.398	18.902
BTP-eC9	89.75	78.61	6.065	18.425	24.489
As-cast	98.90	90.61	3.423	16.428	19.851
BHJ	98.23	87.47	4.781	15.139	19.920
PBHJ	92.04	84.33	4.037	19.575	23.612

Table S2 *J-V* characteristics of PM6:eC9 PBHJ devices with different dilution ratios.

Systems	D:A	V_{oc} [V]	J_{sc} [mA cm^{-2}]	FF [%]	PCE [%]
PM6:eC9	1:0.6/1:1.8	0.851	27.07	78.60	18.10
	1:0.8/1:1.6	0.852	27.45	79.01	18.48
	1:1.0/1:1.4	0.850	26.52	78.33	17.65
	1:1.2/1:1.2	0.850	25.95	77.77	17.15

Table S3 The photovoltaic performance of binary PSCs reported in the literature.

Processing methods	Devices	V_{oc} (V)	J_{sc} (mA cm-	FF (%)	PCE (%)	Refs
BHJ	PBQx-TF:DT-Y6 (Tol)	0.840	26.88	79.74	18.01	[12]
LbL	PBQx-TF/DT-Y6 (o-XY/Tol)	0.839	27.27	80.01	18.30	
BHJ	PM6:Y6 (CF)	0.839	25.97	0.75	16.34	[13]
PBHJ	PM6:Y6 (CF)	0.843	26.63	0.79	17.73	

BHJ	PTQ10:Y6 (CF)	0.850	26.04	0.76	16.82	
PBHJ	PTQ10:Y6 (CF)	0.855	26.70	0.78	17.81	
BHJ	PM6:L8-BO (CF)	0.89	25.87	78.6	18.10	
Q-PHJ	PM6/L8-BO (CB/CF)	0.89	25.62	75.5	17.21	[14]
IHJ	PM6/L8-BO (CB/CF)	0.89	26.11	80.6	18.74	
P-BHJ	PM6:BO-4Cl/PM6:L8-BO	0.907	24.65	79.04	17.67	
T-BHJ	PM6:BO-4Cl:L8-BO (CF)	0.884	26.71	78.26	18.48	[15]
P-PHJ	PM6:BO-4Cl/PM6:L8-BO	0.902	26.98	79.41	19.32	
Green solvent (Anisole:LM)	PBNT-TzTz:Y6-BO (BHJ)	0.88	25.42	0.70	15.65	[16]
Solvent (CF:DIO)		0.84	25.9	72.0	15.9	
Green solvent (Eu:Tet)		0.82	25.7	72.0	15.7	
Green solvent (Lim:Ind)	PM6:BTP:eC9 (BHJ)	0.81	22.8	50.0	10.5	[17]
Green solvent (Pin:EPS)		0.82	24.2	54.0	11.9	
Green solvent (Men:Tet)		0.79	20.8	58.0	10.3	
Solvent (CB)	PM6:BO-4F (PHJ)	0.82	26.2	72.3	15.6	[18]
Green solvent (*o*-XY)	PM6:BO-4F (PHJ)	0.82	26.2	74.3	16.0	
Green solvent (*o*-XY)	PM6:BO-4Cl (BHJ)	0.847	26.86	79.63	18.12	[11]
	PM6:BTP:eC9 (BHJ)	0.847	27.22	80.31	18.52	
Green solvent (*o*-XY)	PBQx-TCl:FF24-Cl (BHJ)	0.886	23.24	74.82	15.40	[19]
	PBQx-TCl:FM24-Cl (BHJ)	0.905	25.66	78.85	18.30	
	PBQx-TCl:MM24-Cl (BHJ)	0.911	25.13	75.84	17.35	

Green solvent (o-XY)	PM6:BTP:eC9 (BHJ)	0.851	26.93	77.48	17.76	**This work**
	PM6:BTP:eC9 (PBHJ)	0.852	27.45	79.01	18.48	

Table S4 Hole- and electron-only mobility of devices with different active layer structures.

**Systems**	μ_h **(cm^2 V^{-1} s^{-1})**	μ_e **(cm^2 V^{-1} s^{-1})**	μ_h **/**μ_e
As-cast	3.80×10^{-4}	4.81×10^{-4}	1.263
BHJ	4.65×10^{-4}	5.59×10^{-4}	1.202
PBHJ	5.10×10^{-4}	5.71×10^{-4}	1.120

Table S5 The key parameters of the photocurrent versus effective voltage with different active layer devices based on PM6:eC9 system.

**Systems**	J_{sat} **[mA cm^{-2}]**	J_{ph}^* **[mA cm^{-2}]**	J_{ph}^{∞} **[mA cm^{-2}]**	J_{ph}^*/J_{sat} **(**η_{diss}**) [%]**	J_{ph}^{∞}/J_{sat} **(**η_{coll}**) [%]**
As-cast	28.76	27.85	25.48	96.72	88.57
BHJ	26.61	26.17	24.01	98.30	90.22
PBHJ	28.44	27.58	25.81	96.99	90.75

Table S6 The key parameters of τ_{ext}, τ_{pho}, and μ for the devices with the different active layers based on PM6:eC9 system.

**Systems**	τ_{ext} **[**μ**s]**	τ_{pho} **[**μ**s]**	μ **[10^{-4} cm^2 V^{-1} s^{-1}]**
As-cast	0.26	8.77	1.66×10^{-4}
BHJ	0.25	13.17	1.89×10^{-4}
PBHJ	0.23	16.65	2.00×10^{-4}

Reference

[1] Lu, G., Shen, Z., Wang, H., Bu, L., & Lu, G. Optical interference on the measurement of film-depth-dependent light absorption spectroscopy and a

correction approach. *Review of Scientific Instruments*, **2023**, 94(2).
[2] Shen, Z., Yu, J., Lu, G., Wu, K., Wang, Q., Bu, L., ... & Lu, G. Surface crystallinity enhancement in organic solar cells induced by spinodal demixing of acceptors and additives. *Energy & Environmental Science*, **2023**, 16(7), 2945-2956.
[3] Yu, J., Shen, Z., Lu, W., Zhu, Y., Liu, Y. X., Neher, D., ... & Lu, G. Composition waves in solution-processed organic films and its propagations from kinetically frozen surface mesophases. *Advanced Functional Materials*, **2023**, 33(40), 2302089.
[4] Becke, A. D. Density-functional exchange-energy approximation with correct asymptotic behavior. *Physical review A*, **1988**, 38(6), 3098.
[5] Lee, C., Yang, W., & Parr, R. G. Development of the Colle-Salvetti correlation-energy formula into a functional of the electron density. *Physical review B*, **1988**, 37(2), 785.
[6] Petersson, A., Bennett, A., Tensfeldt, T. G., Al-Laham, M. A., Shirley, W. A., & Mantzaris, J. A complete basis set model chemistry. I. The total energies of closed-shell atoms and hydrides of the first-row elements. *The Journal of chemical physics*, **1988**, 89(4), 2193-2218.
[7] Marenich, A. V., Cramer, C. J., & Truhlar, D. G. Universal solvation model based on solute electron density and on a continuum model of the solvent defined by the bulk dielectric constant and atomic surface tensions. *The Journal of Physical Chemistry B*, **2009**, 113(18), 6378-6396.
[8] Lu, T., & Chen, F. Quantitative analysis of molecular surface based on improved Marching Tetrahedra algorithm. *Journal of Molecular Graphics and Modelling*, **2012**, 38, 314-323.
[9] Lu, T., & Chen, F. Multiwfn: A multifunctional wavefunction analyzer. *Journal of computational chemistry*, **2012**, 33(5), 580-592.
[10] Cui, Y., Yao, H., Zhang, J., Xian, K., Zhang, T., Hong, L., ... & Hou, J. Single-junction organic photovoltaic cells with approaching 18% efficiency. *Advanced Materials*, **2020**, 32(19), 1908205.
[11] Yang, C., Jiang, M., Wang, S., Zhang, B., Mao, P., Woo, H. Y., ... & An, Q. Hot-casting strategy empowers high-boiling solvent-processed organic solar

cells with over 18.5% efficiency. *Advanced Materials*, **2024**, 36(3), 2305356.
[12] Zhao, J., Zhang, J., Dou, Y., Zhang, K., Zhu, C., Zhong, Z. and Huang, F., 2024. High-performance organic solar cells enabled by the pin structure and ternary strategy. *Journal of Materials Chemistry A*, *12*, pp.24862-24871.
[13] Li, S., Shi, C., Luo, X., Li, D., Lu, X., Hu, Y., Yuan, J. and Zou, Y., 2023. High-Efficiency Binary Organic Solar Cells Enabled by Pseudo-Bilayer Configuration in Dilute Solution. *Solar Rrl*, *7*(9), p.2201090.
[14] Xu, X., Yu, L., Meng, H., Dai, L., Yan, H., Li, R. and Peng, Q., 2022. Polymer solar cells with 18.74% efficiency: from bulk heterojunction to interdigitated bulk heterojunction. Advanced Functional Materials, 32(4), p.2108797.
[15] Wen, L., Mao, H., Zhang, L., Zhang, J., Qin, Z., Tan, L. and Chen, Y., 2024. Achieving Desired Pseudo-Planar Heterojunction Organic Solar Cells via Binary-Dilution Strategy. *Advanced Materials*, *36*(3), p.2308159.
[16] Pang, S., Chen, Z., Li, J., Chen, Y., Liu, Z., Wu, H., Duan, C., Huang, F. and Cao, Y., 2023. High-efficiency organic solar cells processed from a real green solvent. *Materials Horizons*, *10*(2), pp.473-482.
[17] Corzo, D., Rosas-Villalva, D., Tostado-Blázquez, G., Alexandre, E.B., Hernandez, L.H., Han, J., Xu, H., Babics, M., De Wolf, S. and Baran, D., 2023. High-performing organic electronics using terpene green solvents from renewable feedstocks. Nature Energy, 8(1), pp.62-73.
[18] Wan, J., Zeng, L., Liao, X., Chen, Z., Liu, S., Zhu, P., Zhu, H. and Chen, Y., 2022. All-green solvent-processed planar heterojunction organic solar cells with outstanding power conversion efficiency of 16%. Advanced Functional Materials, 32(5), p.2107567
[19] Gong, Y., Zou, T., Li, X., Qin, S., Sun, G., Liang, T., Zhou, R., Zhang, J., Zhang, J., Meng, L. and Wei, Z., 2024. C-shaped ortho-benzodipyrrole-based acceptors with different electronic effects of top substituents for as-cast green-solvent processed high-performance organic solar cells. Energy & Environmental Science, 17(18), pp.6844-6855.
